# Response to comment by Loiselle & Ramchandra (2015)

**DOI:** 10.1098/rsos.150319

**Published:** 2015-08-19

**Authors:** David Colquhoun

I thank Loiselle and Ramchandra (hereafter ‘the authors’) for their interest.

First, I should say that it might have been a mistake to use the term ‘false discovery rate’. This is so because the same term is used in the world of multiple comparison studies. It is defined there in the same way as in my paper, but the context is quite different. Corrections for multiple comparisons all give simply the *p*-value if applied to a single experiment, so they take no account of the false-positive rate that is discussed in my paper. To reduce this source of confusion, it might have been better if I had used the term ‘false-positive rate’ rather than ‘false discovery rate’. However, for the sake of consistency, I will stick to the latter term here.

I should start by reiterating the question that I was aiming to answer. How should one interpret the observation of *p*=0.047 in a single experiment? The conclusion was that, under reasonable assumptions, in at least 26% of such cases, the null hypothesis would be true, so you would be wrong if you claimed to have discovered an effect. The actual rate of false discoveries may be much worse than this, because of *p*-hacking, lack of randomization and all the other suspects.

The authors say

We find the numeric value of 0.1 for the parameter describing ‘the probability that the putative effect is real’ to be wholly unrealistic for divining the appropriate *p*-value to be used as the basis for deciding whether the outcome of an experiment provides evidence ‘for’ or ‘against’ rejection of the null hypothesis.

I could not agree more. The value of 0.1 just happens to be the one that was used for the first example. As the authors say, sometimes it will be too low. In other cases, it may be too high.

The authors' comment points out, quite correctly, that the prevalence of true effects is not generally known. It is obvious that if most of the hypotheses that you test are true, there will not be many false positives (as I pointed out at the end of §6). That is all that is shown in figure 1 of Loiselle & Ramchandra.

The main problem with their figure 1 is that it is based on looking at all tests that give *p*<0.05. Admittedly, that is how I introduced the problem in my figure 2. That is because my figure 2 provides a very simple way of showing that there can be a problem. But my main conclusions are based on the idea that if you have observed *p*=0.047 you should not look at all *p*<0.047, but only at *p*-values close to 0.047. If one accepts that, then the authors' figure 1 becomes irrelevant. It remains true that for *any* prevalence of true effects less than 0.5, the minimum false discovery rate is 26%.

The authors' most substantial argument is that prevalences greater than 0.5 may be legitimate, and if this were true, then of course there would be fewer false discoveries. I cannot say that I find it at all convincing to say that we asked a few co-workers who said that their hypotheses were usually borne out. As the authors themselves say, this argument is circular.

The published papers that the authors looked at often had quite small *p*-values. That would be relevant if one were trying to estimate a global false discovery rate, but it is irrelevant to the question that I was trying to answer.

To postulate a prevalence greater than 0.5 is tantamount to saying that you were confident that your hypothesis was right before you did the experiment. That would imply that it is legitimate to claim that you have made a discovery on the basis of a statistical argument the premise of which is that it is more likely than not that your hypothesis was right before you did the experiment. I suspect that any such argument would be totally unacceptable to reviewers and editors. Certainly, I have never seen a paper that attempted to justify its conclusions in that way.

In the supplementary material, the authors' figure S1 confirms the value of 26% for the false discovery rate when one looks only at 0.045<*p*<0.05. Obviously, that gets smaller for lower *p*-values, as I pointed out in my sections 11, A5 and table 1. As stated at the start, my paper addressed only the question of how to interpret a single observation of *p*=0.047. As the authors have shown, the program I supplied allows any other values to be examined.

The final point in the electronic supplementary material is that the conclusions depend on the effect size. That is, I think, not true if one keeps the power constant. For example, if the true effect size is reduced from 1 to 0.5 s.d., then to keep the power at 0.8 the sample size has to be increased from 16 to 63. Running the example with these numbers gives a false positive rate of 26%, as before, for a prior prevalence of 0.5 (and much worse for lower prevalences).

It should be said that Senn has pointed out that certain sorts of prior distribution can result in false positive rates that are quite close to the *p*-values, for one-sided tests (see slides 10 and 20 in 1 and discussion in 2). This, however, answers a question that is different from that which I am trying to answer 3. I have never seen the smooth Laplacian prior invoked in an experimental study, and, as an experimenter, the point null is what I want to test. I want to be able to eliminate the possibility that the two treatments being compared are identical. If I can convince myself that the treatments are not identical, I then estimate the effect size and try to judge whether it is large enough to matter in practice.

I remain, therefore, convinced that *p*-values, as they are usually used in practice, will lead to many false discoveries.
